# Lactic Acid Bacteria Exert a Hepatoprotective Effect against Ethanol-Induced Liver Injury in HepG2 Cells

**DOI:** 10.3390/microorganisms9091844

**Published:** 2021-08-31

**Authors:** Ji Yeon Lee, Hyemin Kim, Yulah Jeong, Chang-Ho Kang

**Affiliations:** MEDIOGEN, Co., Ltd., Biovalley 1-ro, Jecheon-si 27159, Korea; ljy341@naver.com (J.Y.L.); astrongirl.hm@gmail.com (H.K.); jinhwa110931@gmail.com (Y.J.)

**Keywords:** probiotics, oxidative stress, alcohol, hepatoprotective, CYP2E1

## Abstract

Alcoholic liver fatty disease (ALFD) is caused by excessive and chronic alcohol consumption. Alcohol consumption causes an imbalance in the intestinal microflora, leading to liver disease induced by the excessive release of endotoxins into the hepatic portal vein. Therefore, research on the intestinal microflora to identify treatments for ALFD is increasing. In this study, the protective effects of lactic acid bacteria (LAB) strains, including *Levilactobacillus brevis, Limosilactobacillus reuteri*, and *Limosilactobacillus fermentum*, were evaluated in ethanol-induced HepG2 cells. Among the evaluated LAB, nine strains increased aldehyde dehydrogenase (ALDH) levels and downregulated lipid peroxidation and liver transferase in the ethanol-induced HepG2 cells. Moreover, *L. brevis* MG5280 and MG5311, *L. reuteri* MG5458, and *L. fermentum* MG4237 and MG4294 protected against ethanol-induced HepG2 cell damage by regulating *CYP2E1*, antioxidant enzymes (*SOD*, *CAT*, and *GPX*), lipid synthesis factors (*SREBP1C* and *FAS*), and lipid oxidation factors (*PPARα*, *ACO*, and *CPT-1*). Moreover, five LAB were confirmed to be safe probiotics based on antibiotic susceptibility and hemolysis assays; their stability and adhesion ability in the gastrointestinal tract were also established. In conclusion, *L. brevis* MG5280 and MG5311, *L. reuteri* MG5458, and *L. fermentum* MG4237 and MG4294 may be useful as new probiotic candidates for ALFD prevention.

## 1. Introduction

Alcoholic liver disease (ALD) is caused by chronic alcohol consumption and includes alcohol-induced liver cirrhosis, fibrosis, hepatitis, and liver cancer [1,2]. In particular, alcoholic fatty liver disease (AFLD), a common liver disease in many countries, is responsible for the death of at least three million people according to the World Health Organization (WHO) [3]. Three-month short-term mortality rate in patients with severe alcoholic steatohepatitis is very high, approaching 40–50% [4]. As only a few treatments are available for AFLD, the discovery of new useful treatments for AFLD is needed [2].

Alcohol is mainly metabolized via oxidation, catalyzed by alcohol dehydrogenase (ADH) and aldehyde dehydrogenase (ALDH) [5]. In this metabolism, cytochrome P450 2E1 (CYP2E1), which is activated in conjunction with ADH, induces oxidative stress, causing an imbalance between the production and elimination of reactive oxygen species (ROS) [6]. However, ROS levels are reduced by the expression of antioxidant enzymes, including superoxide dismutase (SOD), catalase (CAT), and glutathione peroxidase (GPX) [7]. Alcohol intake also delays fatty acid oxidation by inhibiting peroxisome proliferator-activated receptor α (PPARα) and increases lipogenesis by activating sterol regulatory element-binding transcription factor 1C (SREBP1C), which may lead to fatty liver [1]. Therefore, a functional food that exhibits antioxidant activity and modulates lipid metabolism in hepatocytes could be a therapeutic agent for preventing AFLD.

Lactic acid bacteria (LAB), the most commonly used probiotics, are living microorganisms that provide health benefits by improving the balance in the host’s intestinal microbiota [8]. Recently, LAB, especially the *Lactobacilaceae* family, have proven to be therapeutic based on scientific research that revealed their range of health benefits, including diarrhea prevention, anti-allergy effects, and immune system modulation [9]. LAB can prevent AFLD by suppressing oxidative stress and improving the intestinal barrier function to reduce endotoxemia in the gut–liver axis [10]. For example, the amount of *Bacteroides* and *Firmicutes* is low in the gut microbiota of patients with ALD, leading to intestinal dysbiosis and pathogenic bacterial overgrowth. However, LAB can normalize the intestinal microflora [2]. In a previous study, *Levilactobacillus brevis* HY7410 and *Limosilactobacillus fermentum* MG590 lowered blood alcohol concentration by enhancing ADH and ALDH activity [11,12]. Moreover, *L. brevis* SBC8803, *Limosilactobacillus reuteri* DSM17938, and *L. fermentum* protected the liver of ethanol-fed mice [3,13,14]. Thus, LAB modulate the altered gut microbiota caused by alcohol and could thus be a promising treatment for the prevention of ALD. However, studies on the protective effects of LAB on AFLD are insufficient compared with those on non-AFLD.

Thus, we determined the ALDH activity and antioxidant and lipid metabolism of LAB, including *L. brevis*, *L. reuteri*, and *L. fermentum* isolated from humans and fermented food, in ethanol-induced HepG2 cells. Additionally, to confirm the properties of the probiotics, the safety and intestinal cell adhesion ability of those LAB having an ALFD inhibitory effect were determined.

## 2. Materials and Methods

### 2.1. Isolation of Bacterial Strains and Preparation of Cell-Free Extracts (CFEs)

All LAB strains used in this study were collected from MEDIOGEN (Jecheon, Korea). LAB used in this study were isolated from humans (MG4229, MG4296, MG4224, MG4231, MG4237, MG4244, MG4294, and MG4295) and fermented foods (MG5250, MG5280, MG5306, MG5311, MG5025, MG5149, and MG5458). In addition, *L. fermentum* MG590, a probiotic that alleviates AFLD, was used as a positive control [12]. All strains were cultured in MRS broth (de Man, Rogosa and Sharpe; Difco, Detroit, MI, USA) for 18 h at 37 °C under anaerobic chamber (Hanbaek Scientific Co., Gyeonggi-do, Korea).

CFEs were prepared according to the method of Park et al. [15]. For CFEs, each strain was collected by centrifugation (4000× *g*, 20 min at 4 °C). The collected pellet was lyophilized and resuspended in phosphate-buffered saline (PBS) at 10 mg/mL. The suspension was homogenized for 50 s using a sonicator (KFS-150N; Korea Process Technology Ltd., Seoul, Korea) and allowed to rest on ice for 1 min (repeated three times); the suspension was then centrifuged (4000× *g*) for 10 min at 4 °C. The supernatants were filter-sterilized using a 0.22 µm polytetrafluoroethylene membrane filter (ADVANTEC, Tokyo, Japan) and kept at −80 °C until use.

### 2.2. ALDH Activity

ALDH activity was determined as previously described [16]. In 10 µL of CFEs, 700 µL of distilled water, 375 µL of 1 M Tris–HCl buffer (pH 8.8, Sigma–Aldrich, St. Louis, MO, USA), and 150 µL of 25 mM NAD^+^ (Sigma–Aldrich) were reacted. After 10 min, ALDH (5 U/mL, Sigma–Aldrich) was added to the mixed samples. The optical density in kinetic mode (interval 10 min for 90 min) was determined at 340 nm using a microplate reader (EPOCH2, Biotek, Winooski, VT, USA). The protein content of the CFEs was determined using the Bradford assay (Bio-Rad, Hercules, CA, USA). The ALDH activity was calculated as the molar extinction coefficient of NADH (6.22 mM^−1^ cm^−1^) and expressed as units/mg protein/min.

### 2.3. Cell Culture

HepG2 cells (88065, KCLB, Seoul, Korea) were cultured in minimum essential media (MEM; Gibco, MT, USA) with 10% fetal bovine serum (FBS; Gibco) and 1% penicillin–streptomycin (PS; Gibco). HT-29 cells (30038, KCLB) were cultured in Dulbecco’s modified Eagle’s medium (DMEM; Gibco) with 10% FBS and 1% PS at 37 °C in a 5% CO_2_ incubator. The cells were subcultured at 70%–80% confluence.

### 2.4. Cell Viability

Cell viability was determined using the 3-(4,5-dimethylthiazol-2-yl)-2,5-diphenyltetrazolium bromide (MTT) assay [17]. HepG2 cells were seeded in 96-well plates at 4 × 10^5^ cells/mL. After overnight growth, the cells were treated with CFEs for 1 h and then with or without ethanol for the next 24 h. The MTT solution (0.2 mg/mL) was added, and the cells were further cultured for 2–4 h. After incubation, the formazan crystals in each well were dissolved in DMSO. The absorbance at 550 nm was measured using a microplate reader.

### 2.5. Determination of Lipid Peroxidation and Glutathione (GSH) Content

Measurement of lipid peroxidation and GSH content was performed according to the manufacturer’s instructions (Cayman Chemical, Ann Arbor, MI, USA) and normalized to protein content using the Bradford assay.

For evaluation of lipid peroxidation, 5 × 10^6^ cells in 100 mm plates were incubated in the presence or absence of CFEs for 1 h. Thereafter, the cells were stimulated with ethanol (3%) for 24 h. After incubation, lipid peroxidation was measured using a microplate reader at 540 nm and calculated using the malondialdehyde (MDA) calibration curve.

To measure GSH content, the cells (4 × 10^5^ cells/mL) in a 6-well plate were incubated with or without CFEs for 1 h and then stimulated with ethanol (3%) for 24 h. After incubation, total glutathione was measured using a microplate reader at 405 nm.

### 2.6. Measurement of Liver Injury

Liver injury was measured by alanine aminotransferase (ALT) and aspartate aminotransferase (AST) levels using commercially available assay kits (Cayman Chemical) in accordance with the manufacturer’s instructions and normalized to protein content using the Bradford assay. Briefly, cells (5 × 10^5^ cells/mL) in a 6-well plate were incubated in the presence or absence of CFEs for 1 h. Thereafter, the cells were stimulated with 3% ethanol for 24 h. After incubation, the ALT and AST levels of the cell lysates were measured using a microplate reader at 340 nm.

### 2.7. mRNA Extraction and Quantitative Real Time-Polymerase Chain Reaction (qRT-PCR)

The mRNA from HepG2 cells was isolated using 0.5 mL of NuceloZOL (MACHEREY–NAGEL GmbH & Co. KG, Dueren, Germany), according to the manufacturer’s instructions. cDNA was synthesized from 1 μg of total RNA using reverse transcriptase premix (Intron, Seongnam-si, Korea). qRT-PCR was performed using the CFX Connect Real-Time PCR Detection System (Bio-Rad), and target gene expression was assessed using the iQ™ SYBR^®^ Green Supermix (Bio-Rad) by the standard metho (95 °C for 3 min, followed by 39 cycles at 95 °C for 10 s, 55–60 °C for 30 s, 72 °C for 30 s). The forward and reverse primers used are listed in Table S1. The relative expression of the target gene was normalized to that of *GAPDH* and analyzed by the 2^−ΔΔCT^ method.

### 2.8. Probiotic Properties

#### 2.8.1. Antibiotic Susceptibility and Hemolysis Assay

Antibiotic susceptibility was measured using antibiotic strips according to the manufacturer’s instructions (bioMérieux, Marcy-l’Étoile, France). Antibiotic resistance was confirmed according to the European Food Safety Authority (EFSA) guidelines [18].

The hemolysis assay was performed using tryptic soy agar (BD Bioscience, NJ, USA) plates containing 5% (*w*/*v*) sheep blood (MBCell, Seoul, Korea) [19]. The zone was observed as a green colony (α-hemolysis), a clean zone (β-hemolysis), and no color change (γ-hemolysis).

#### 2.8.2. Gastrointestinal Tract (GIT) Stability and Adhesion

The survival rate in simulated GIT was evaluated according to the Maragkoudakis’s method with slight modifications [20]. Briefly, the LAB were cultured for 18 h and washed twice with PBS (pH 7.4) after centrifugation (4000× *g* for 5 min at 4 °C). The collected LAB were resuspended to 10^8^ CFU/mL in simulated gastric fluid containing 3 g/L pepsin (adjusted to pH 3 and 4 with 1 N HCl) for 2 h and simulated intestinal fluid containing 1 g/L pancreatin adjusted to pH 7 and 8 with 1 N NaOH for 4 h, incubated at 37 °C. LAB were measured by counting viable cells using MRS agar plates.

The adhesion ability of LAB was evaluated using HT-29 colorectal cells, as described previously [21]. HT-29 colorectal cells (1 × 10^5^ cells/mL) were incubated in 12-well plates in 5% CO_2_ at 37 °C for 24 h. The LAB were cultured in MRS broth at 37 °C for 24 h. The strains were resuspended at 1 × 10^8^ CFU/mL in DMEM without FBS and PS and administered to cells. After 2 h, the cells were washed twice and then detached with PBS. The number of viable LAB was measured by plate counting on MRS agar and calculated by log CFU/mL.

### 2.9. Statistical Analysis

All experimental results are presented as the mean ± standard deviation (SD, *n* = 3). The statistical significance of differences was calculated using one-way analysis of variance (ANOVA) followed by Duncan’s multiple range test at *p* < 0.05 (SPSS, version 21; IBM Inc., Armonk, NY, USA).

## 3. Results

### 3.1. ALDH Activity of the LAB Strains

**Figure 1:**
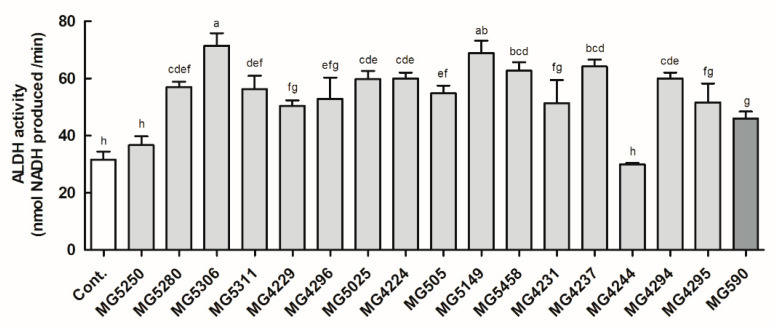
Effect of CFEs (5 mg/mL) from LAB strains on ALDH activity. Data are expressed as the mean ± SD (*n* = 3). Different letters above the columns indicate significance at *p* < 0.05 based on Duncan’s test. Cont, control.

ALDH activity of all LAB strains, except MG5250 and MG4244, was increased compared with that of the control (Figure 1). In addition, nine LAB strains—MG5280 (1.83-fold of the control), MG5306 (2.29-fold of the control), MG5311 (1.80-fold of the control), MG4224 0 (1.60-fold of the control), MG505 (1.73-fold of the control), MG5149 (2.21-fold of the control), MG5458 (2.01-fold of the control), MG4237 (2.06-fold of the control), and MG4294 (1.92-fold of the control)—showed higher activity than MG590 (1.48-fold of the control), which was used as a positive control. Therefore, nine LAB strains with higher ALDH activity than the positive control were tested in HepG2 cells.

### 3.2. Protective Effect of LAB Strains on Ethanol-Induced HepG2 Cells

Prior to the assessment, the cytotoxic and protective effects of CFEs from LAB were confirmed in HepG2 cells with or without ethanol (Table 1). All LAB strains showed no cytotoxicity at 100 μg/mL (91.14 to 100.89%) in the HepG2 cells. After treatment with various concentrations of ethanol to induce cell injury, a significant cell death of less than 60% was confirmed when the HepG2 cells were stimulated with >3% ethanol (Figure S1). Thus, in subsequent experiments, HepG2 cell injury was induced by treatment with 3% ethanol. Viability of the ethanol-induced HepG2 cells was decreased by approximately 55% when compared with that of the non-ethanol-treated control. Nonetheless, all LAB strains were found to increase the viability of HepG2 cells treated with ethanol (73.27 to 91.47%).

**Figure 2:**
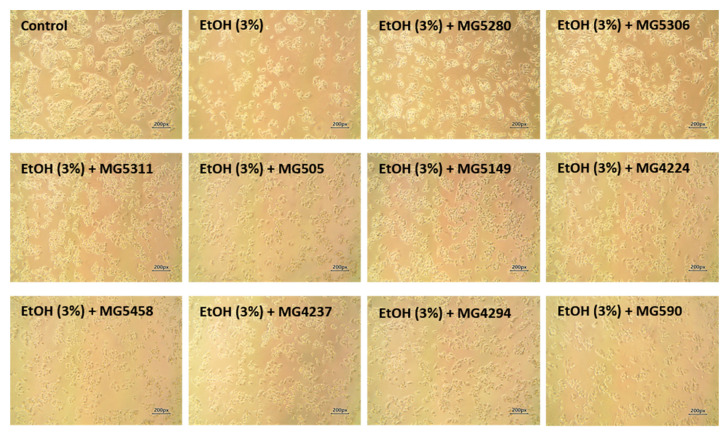
Microscopic morphological images of ethanol-induced HepG2 cells treated with or without CFEs (100 μg/mL) from LAB strains. The images were captured at 10× magnification by phase-contrast light microscopy. EtOH, ethanol.

In terms of cell morphology, treatment of HepG2 cells with ethanol resulted in a change in epithelial cell shape and number (Figure 2). However, pretreatment with LAB strains averted cell damage caused by ethanol treatment by maintaining the original cell shape and number.

### 3.3. LAB Strains Regulate Oxidative Stress in Ethanol-Induced HepG2 Cells

**Figure 3:**
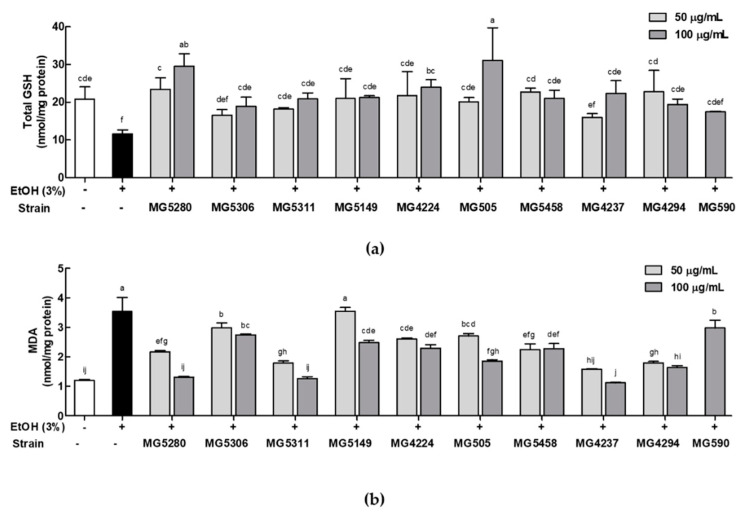
Effects of CFEs from LAB strains on (**a**) total GSH and (**b**) lipid peroxidation (determined by MDA levels) in ethanol-induced HepG2 cells. Data are expressed as mean ± SD (*n* = 3). Different letters at the column indicate significance at *p* < 0.05 based on Duncan’s test. EtOH, Ethanol.

To confirm the protective effect of LAB against oxidative damage induced by ethanol treatment in HepG2 cells, total GSH and lipid peroxidation were measured. GSH content in cells induced by ethanol decreased by 0.56-fold when compared with that in cells induced by the control; however, when treated with LAB strains, GSH content was similar to or greater than that induced by the control (Figure 3a). In particular, MG5280 (29.50 ± 3.39 nmol/mg protein) and MG505 (31.04 ± 8.66 nmol/mg protein) showed significantly higher activity than the positive control MG590 (20.91 ± 0.45 nmol/mg protein).

Lipid peroxidation in HepG2 cells induced by ethanol was indicated by MDA levels (Figure 3b). The MDA levels of HepG2 cells treated only with ethanol was significantly increased by 2.94-fold compared with those in cells treated with the control; however, when pretreated with the nine LAB strains, the MDA levels of HepG2 cells were significantly decreased by approximately 0.38- to 0.99-fold compared with those of cells treated with the positive control *L. fermentum* MG590. Additionally, *L. brevis* MG5280 (1.31 ± 0.05 nmol/mg protein), *L. brevis* MG5311 (1.27 ± 0.12 nmol/mg protein), and *L. fermentum* MG4237 (1.12 ± 0.04 nmol/mg protein) markedly improved MDA levels as much as the control (1.20 ± 0.06 nmol/mg protein).

### 3.4. LAB Strains Protect against Liver Injury Induced by Ethanol in HepG2 Cells

**Figure 4:**
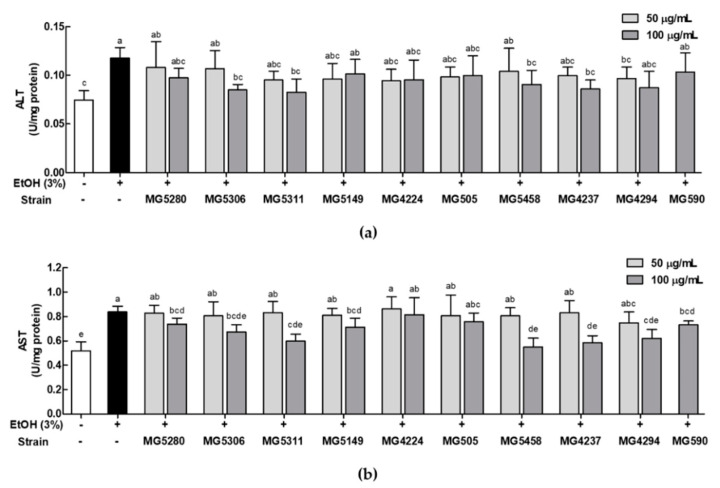
Inhibition of (**a**) ALT and (**b**) AST levels by CFEs from LAB strains in ethanol-induced HepG2 cells. Data are expressed as mean ± SD (*n* = 3). Different letters on the column indicate significance at *p* < 0.05 based on Duncan’s test. EtOH, ethanol.

The protective effect of LAB strains against liver injury was assessed by the ALT and AST levels in ethanol-induced HepG2 cells. Ethanol significantly increased the levels of ALT and AST by 1.58- and 1.61-fold, respectively, compared with the control (Figure 4a,b). However, these increases were reduced upon treatment with the LAB strains by 0.66 to 0.99-fold in ALT and 0.70 to 0.92-fold in AST when compared with the ALT and AST levels in ethanol-induced HepG2 cells. In particular, *L. brevis* MG5311 (0.08 ± 0.01 and 0.60 ± 0.06 U/mg protein), *L. reuteri* MG5458 (0.09 ± 0.01 and 0.55 ± 0.07 U/mg protein), and *L. fermentum* MG4237 (0.09 ± 0.02 and 0.58 ± 0.06 U/mg protein) significantly suppressed the enzyme levels (ALT and AST, respectively) in HepG2 cells treated with ethanol relative to the control (0.07 ± 0.01 and 0.52 ± 0.07 U/mg protein).

Based on these results, *L. brevis* MG5280 and MG5311, *L. reuteri* MG5458, and *L. fermentum* MG4237 and MG4294 were selected as potential probiotics that can reduce damage to ethanol-induced HepG2 cells. Therefore, it was confirmed that these LAB had a protective effect against ALD. We then aimed to identify the underlying mRNA regulation related with ALFD.

### 3.5. LAB Strains Modulate Ethanol Metabolism by Enhancing mRNA Expression of Antioxidant Enzyme and Lipid Metabolism in Ethanol-Induced HepG2 Cells

**Figure 5:**
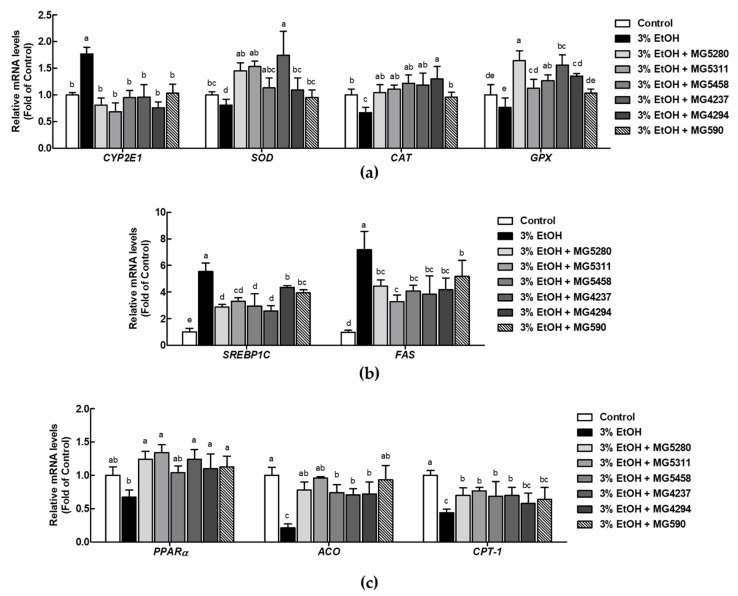
Effect of CFEs (100 μg/mL) from LAB strains on mRNA expression of (**a**) *CYP2E1* and antioxidant enzymes (*SOD*, *CAT* and *GPX*), (**b**) lipid synthesis factors (*SREBP1C* and *FAS*), and (**c**) lipid oxidation factors (*PPARα*, *ACO* and *CPT-1*). Data are expressed as the mean ± SD (*n* = 3). Different letters on the column indicate significance at *p* < 0.05 based on Duncan’s test. EtOH, ethanol.

The effect of the LAB strains selected for the experiment on AFLD-related mRNA expression was investigated. Under oxidative stress in ethanol-induced HepG2 cells, expression level of *CYP2E1* was significantly increased by 1.77-fold and that of *SOD, CAT*, and *GPX* was significantly reduced by 0.67-, 0.81-, and 0.76-fold, respectively, when compared with that in the control (Figure 5a). LAB treatment remarkably reversed the expression of these mRNA. In addition, to confirm the effect of LAB on ethanol-induced HepG2 cells, mRNA expression related to lipid metabolism was examined. Expression levels of *SREBP1C* and fatty acid synthase (*FAS*), which are lipogenesis-related factors, were increased by 5.54- and 7.18-fold after treatment of HepG2 cells with ethanol when compared with those after treatment with the control; however, LAB displayed a marked inhibition rate that ranged from 0.46 to 0.79-fold (Figure 5b). Expression levels of lipid oxidation factors, including *PPAR*, acyl–CoA oxidase (*ACO*), and carnitine palmitoyltransferase-1 (*CPT-1*), were significantly reduced in ethanol-induced HepG2 cells by 0.21-, 0.67-, and 0.44-fold compared with those in cells induced by the control. As expected, LAB exhibited significant upregulation of all tested lipid oxidation factors, with inhibition rates ranging from 1.34 to 4.50-fold (Figure 5c). In addition, LAB showed a better mRNA expression-modulating effect of all tested factors than *L. fermentum* MG590, the positive control.

Taken together, all LAB have been shown to inactivate CYP2E1 to stimulate the antioxidant enzymes (*SOD*, *CAT*, and *GPX*) and modulate adipose metabolism-related factors (*SREBP1C*, *FAS*, *PPAR*, *ACO*, and *CPT-1*) in ethanol-induced HepG2 cells. *L. brevis* MG5280 and MG5311, *L. reuteri* MG5458, and *L. fermentum* MG4237 and MG4294 were also demonstrated to be effective at restoring ALFD through regulation of antioxidant enzyme expressions and lipid metabolism pathway.

### 3.6. Probiotic Properties of the LAB Strains

#### 3.6.1. Safety of LAB as Probiotics

To confirm whether LAB can be used as a probiotic, safety tests using *L. brevis* MG5280 and MG5311, *L. reuteri* MG5458, and *L. fermentum* MG4237 and MG4294 were performed (Table 2). Antibiotic susceptibility was confirmed using the EFSA minimum inhibitory concentration (MIC) cutoff value [18]. All other antimicrobials, except tetracycline from *L. brevis* MG5311 and erythromycin from *L.* *fermentum* MG4237, were found to be below the standard indicated in Table S2. If the strain has hemolytic activity, the host’s red blood cells are destroyed [19]. By confirming the hemolytic activity, all strains were identified as γ-hemolytic—that is, no hemolytic activity was observed (Figure S2).

#### 3.6.2. GIT Stability and Adhesion on HT-29 Colorectal Cells of *L. brevis* MG5311 and *L. fermentum* MG4237

The properties of *L. brevis* MG5280 and MG5311, *L. reuteri* MG5458, and *L. fermentum* MG4237 and MG4294 as probiotics were assessed in artificial GIT, including their adhesion ability to HT-29 colorectal cells (Table 3). In stimulated GIT, all strains had a survival rate of >98%. The cell number in the stimulated GIT ranged from 7.44 to 7.85 Log CFU/mL; the initial cell number ranged from 7.50 to 7.75 Log CFU/mL. Additionally, all strains could adhere to HT-29 colorectal cells, with an adhesion rate ranging from 55.36 to 84.77%. Among them, *L. brevis* MG5280 (84.21 ± 0.26%) and MG5311 (84.77 ± 0.45%) showed high adhesion rates.

## 4. Discussion

Excessive alcohol consumption leads to liver damage [2]. ALD refers to a broad spectrum of alcohol-induced liver damage, including fatty liver, hepatitis, liver fibrosis, and cirrhosis [5]. Among these diseases, most people who frequently drink alcohol have fatty liver. Thus, the prevalence of ALFD is increasing [2]. The social costs, including the medical expenses of ALFD and its treatment, crimes, and accidents, are significant [22]. Abstinence from alcohol consumption may reverse mild ALD, but no effective drug has been found to treat ALFD [2]. Nonetheless, recent studies have reported that probiotics improve liver function [23]. In ethanol metabolism, ALDH plays an important role in converting highly toxic acetaldehyde decomposed by ADH into acetic acid, and oxidizing acetic acid to carbon dioxide and water, which are harmless to the human body [12]. Probiotics can contribute to ethanol metabolism by secreting their own ALDH to reduce aldehydes [12]. In this study, we demonstrated that LAB showed higher activity in ALDH, downregulated oxidative stress and lipogenesis genes, and upregulated lipid oxidation genes in ethanol-treated HepG2 cells. In addition, LAB, which have a protective function against ethanol-induced HepG2 cells, have been demonstrated to be valuable probiotics. Thus, this study was conducted to demonstrate the preventive efficacy of LAB, which can be used as a health functional food and a therapeutic alternative, against ALFD.

Oxidative stress is one of the factors that significantly contribute to the pathogenesis of ALD [24]. Oxidative stress, particularly ROS, is known to cause the oxidation of unsaturated fatty acids to produce lipid peroxides that induce fatty acid side chain reactions and MDA to damage cell structure, function, and DNA [2]. CYP2E1, which is expressed due to ROS generation, induces ALD and progresses to an advanced disease stage [25]. The increase in these factors is known to be reduced by GSH and antioxidant enzymes, such as SOD, CAT, and GPX, which convert O_2_^−^ to H_2_O [26,27]. Therefore, the inhibition of *CYP2E1* expression and an increase in antioxidant enzymes can effectively block the progression of ALD. By investigating LAB, *L. brevis* MG5280 and MG5311, *L. reuteri* MG5458, *L. fermentum* MG4237, and MG4294 were found to show excellent efficacy at relieving oxidative stress by elevating GSH and antioxidant enzymes. Our results were similar to those obtained with Probiotic V, a product that includes various LAB, which was found to reduce oxidative stress by inhibiting lipid peroxidation and the expression of CYP2E1 in HepG2 cells exposed to ethanol [28]. Moreover, the activity of enzymes, such as AST and ALT, has been reported as one of the most sensitive markers of hepatotoxicity [29]. In this study, the five LAB mentioned above were confirmed to alleviate ethanol-induced hepatotoxicity.

Alcohol consumption can affect lipid metabolism in the liver and cause hepatic steatosis [4]. The early growth response-1 (Erg-1) transcription factor involved in cellular stress is expressed by the aldehyde produced by *CYP2E1*, which stimulates *SREPB-1C*, a transcription factor that regulates hepatic cholesterol metabolism [30]. *SREBP-1C* induces lipid and cholesterol synthesis by promoting *FAS* [31]. Treatment with five LAB led to the inhibition of *SREBP1C* and *FAS* mRNA expression. Our results are consistent with a report by Farhin et al. who revealed that probiotics downregulated srebp1c and FAS in HepG2 cells exposed to ethanol [28]. Chronic alcohol consumption impedes lipid oxidation due to erroneous lysosomal biosynthesis, thereby delaying lipid degradation [30]. The transcription factor, *PPARα,* is increased by alcohol and affects the expression of subfactors *ACO* and *CPT-1*, which contribute to lipid oxidation in the mitochondria [32]. In our study, five LAB upregulated the mRNA expression of PPARα, ACO, and CPT-1 in ethanol-induced HepG2 cells. Hong et al. and Chu et al. reported that LAB treatment enhanced lipid oxidation by increasing the expression of PPARα, ACO, and CPT-1 in HepG2 cells [33,34]. Therefore, *L. brevis* MG5280 and MG5311, *L. reuteri* MG5458, and *L. fermentum* MG4237 and MG4294 could be a strategy to protect against ALFD by regulating lipid synthesis and oxidation in alcohol-induced HepG2 cells.

The term probiotics was derived as a comparative concept for the risk of antibiotics against LAB, which are microorganisms that play a beneficial role in the human body [35]. For LAB to be used as a probiotic, they must undergo safety and stability verification [36]. Thus, to use the five selected LAB as probiotics, safety and stability must be demonstrated. By conducting hemolytic and antibiotic resistance tests to confirm the safety of probiotics, we found that there were no hemolytic properties in any strain. However, *L. brevis* MG5311 was confirmed to be resistant to tetracycline; thus, further studies such as plasmid association are required. As probiotics play a role in maintaining health and regulating human GIT, their ability to grow at low and high pH by pepsin and bile should be confirmed [37]. In this study, all five LABs survived more than 98% in stimulated GIT. HT-29 colorectal cells are used extensively in adhesion studies because they best represent the morphological and physiological properties of human enterocytes [37]. Compared to other LAB, such as *Lacticaseibacillus rhamnosus* GG (10.33%), *Lactiplantibacillus plantarum* AdF10 (12.88%), and *L. reuteri* E (23.83%), the adhesion ability of all strains was significantly higher than 55.36% [38,39]. *L. brevis* MG5280 and MG5311, *L. reuteri* MG5458, and *L. fermentum* MG4237 and MG4294 had better survival and adhesion than the above-mentioned probiotics, suggesting that they can be used as probiotics with improved efficacy. To more conclusively demonstrate the efficacy of reducing ALFD using a probiotic, further studies are needed to determine whether the same efficacy appears in vivo.

## 5. Conclusions

**Figure 6:**
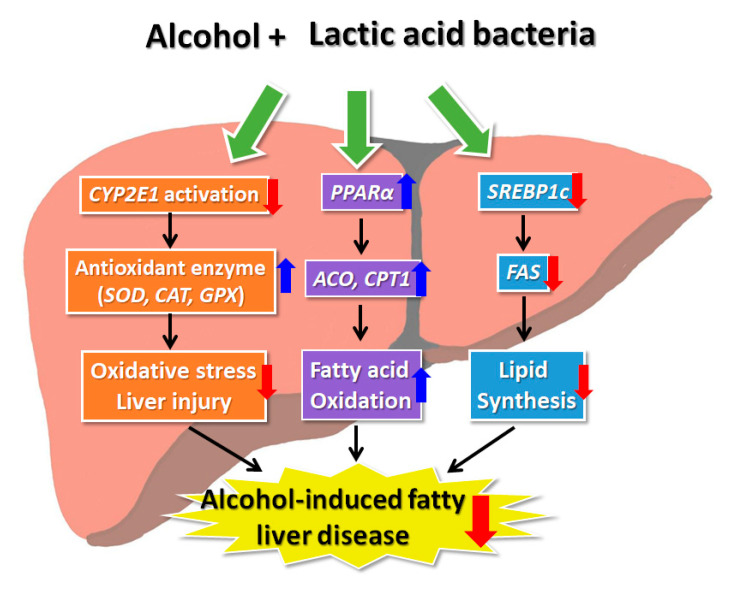
Lactic acid bacteria as probiotics exert a hepatoprotective effect by modulating antioxidant and lipid metabolism in ethanol-induced HepG2 cells.

In conclusion, our findings suggest that LAB are effective at ameliorating damage in ethanol-induced HepG2 cells. In the current study, treatment with LAB reduced the expression level of *CYP2E1* and increased the levels of antioxidant enzymes (*SOD, CAT,* and *GPX*) in ethanol-induced HepG2 cells to prevent cell injury. Further, LAB were found to possess a mechanism that contributes to AFLD prevention by relieving steatohepatitis through the regulation of abnormal lipid metabolism by ethanol (Figure 6). Therefore, LAB, including *L. brevis* MG5280 and MG5311, *L. reuteri* MG5458, and *L. fermentum* MG4237 and MG4294, as probiotics could serve as a functional food and a therapeutic agent for preventing ALFD.

## Figures and Tables

**Table 1:** Effect of CFEs from LAB strains on the viability of HepG2 cells exposed with or without ethanol.

Lactic Acid Bacteria (μg/mL)			Cell Viability (%)	
			Control	+ 3% Ethanol
Untreated			100.00 ± 5.68	55.40 ± 2.07
*Levilactobacillus brevis*	MG5280	50	99.13 ± 7.37	79.99 ± 5.68 ***
		100	97.26 ± 5.94	73.27 ± 4.13 ***
	MG5306	50	96.91 ± 4.38	76.36 ± 3.47 ***
		100	94.43 ± 2.04	75.04 ± 4.24 ***
	MG5311	50	98.17 ± 5.43	83.47 ± 1.53 ***
		100	99.63 ± 3.52	86.66 ± 2.17 ***
*Limosilactobacillus reuteri*	MG4224	50	96.26 ± 5.03	85.02 ± 1.84 ***
		100	99.49 ± 3.76	87.85 ± 0.89 ***
	MG505	50	97.44 ± 5.55	85.02 ± 0.70 ***
		100	96.58 ± 0.53	87.85 ± 1.91 ***
	MG5149	50	92.51 ± 6.83	83.47 ± 8.61 ***
		100	94.72 ± 11.25	86.66 ± 7.77 ***
	MG5458	50	91.26 ± 1.18	82.60 ± 1.31 ***
		100	91.14 ± 6.32	89.59 ± 0.96 ***
*Limosilactobacillus fermentum*	MG4237	50	95.37 ± 2.16	80.33 ± 2.04 ***
		100	91.71 ± 0.46	91.47 ± 1.23 ***
	MG4294	50	93.89 ± 2.90	80.53 ± 1.92 ***
		100	93.89 ± 3.59	80.53 ± 0.84 ***
	MG590	50	100.89 ± 2.31	68.82 ± 1.46 **
		100	100.63 ± 2.29	75.00 ± 2.73 ***

The results are expressed as mean ± SD (*n* = 3). Significance was based on Duncan’s test: ** *p* < 0.01, and *** *p* < 0.001 compared to the same column control.

**Table 2:** Results of antimicrobial test with the LAB strains.

Antimicrobiotics ^1^	*L. brevis*		*L. reuteri*	*L. fermentum*	
	MG5280	MG5311	MG5458	MG4237	MG4294
Ampicillin	S (0.75)	S (0.5)	S (0.75)	S (0.094)	S (0.094)
Gentamicin	S (0.094)	S (0.047)	S (2)	S (0.19)	S (0.19)
Kanamycin	S (3)	S (3)	S (6)	S (4)	S (4)
Streptomycin	S (4)	S (6)	S (24)	S (3)	S (6)
Tetracycline	S (6)	R (>256)	S (2)	S (3)	S (1.5)
Chloramphenicol	S (2)	S (4)	S (3)	S (3)	S (3)
Erythromycin	S (0.047)	S (0.047)	S (0.25)	R (3)	S (0.25)
Clindamycin	S (1.5)	S (2)	S (0.016)	S (0.023)	S (0.016)

^1^ Susceptible (S) and resistant (R) strains according to the microbiology cutoff values from the EFSA guideline [18]. The minimum inhibitory concentrations are indicated in parentheses (µg/mL).

**Table 3:** Tolerance to artificial GI tract and adhesion of *L. brevis* MG5311 and *L. fermentum* MG4237 to HT-29 cells.

Experiment		*L. brevis*		*L. reuteri*	*L. fermentum*	
		MG5280	MG5311	MG5458	MG4237	MG4294
Stimulated gastrointestinal fluid (Log CFU/mL)	Initial	7.66 ± 0.02	7.50 ± 0.04	7.57 ± 0.04	7.75 ± 0.03	7.63 ± 0.01
	pH 3	7.65 ± 0.05	7.70 ± 0.02	7.58 ± 0.07	7.85 ± 0.03	7.71 ± 0.07
	pH 4	7.64 ± 0.01	7.72 ± 0.06	7.58 ± 0.06	7.82 ± 0.08	7.72 ± 0.07
	pH 7	7.65 ± 0.06	7.70 ± 0.04	7.51 ± 0.00	7.83 ± 0.01	7.56 ± 0.07
	pH 8	7.63 ± 0.11	7.66 ± 0.10	7.44 ± 0.06	7.73 ± 0.01	7.55 ± 0.10
Adhesion ability	Initial	8.52 ± 0.04	7.23 ± 0.04	7.23 ± 0.04	7.23 ± 0.04	7.23 ± 0.04
(Log CFU/mL)	Adherent	8.77 ± 0.02	6.96 ± 0.03	6.96 ± 0.03	6.96 ± 0.03	6.96 ± 0.03
Cell adhesion (%)		84.21 ± 0.26	84.77 ± 0.45	70.76 ± 0.865	79.29 ± 0.32	55.36 ± 1.00

Data are expressed as the mean ± SD of duplicate experiments.

## Data Availability

Data are available in a publicly accessible repository/data are contained within the article or Supplementary Materials.

## Supplementary Materials

The following are available online at https://www.mdpi.com/article/10.3390/microorganisms9091844/s1, Table S1: Primers sequence used for qRT-PCR.; Table S2: The minimum inhibitory concentrations (MIC) cut-off value from EFSA. Figure S1: The viability of ethanol (EtOH) on HepG2 cells; Figure S2: The hemolytic activity of LAB strains.
