# Supplementary material for: Lactic Acid Bacteria Exert a Hepatoprotective Effect against Ethanol-Induced Liver Injury in HepG2 Cells
Source: Microorganisms. 2021 Aug 31;9(9):1844. doi: 10.3390/microorganisms9091844 (PMC8465258; doi:10.3390/microorganisms9091844)
Supplement: Supplementary file 1 [file microorganisms-09-01844-s001.zip › microorganisms-1358365-supplementary.pdf]

Table S1. Primers sequence used for qRT-PCR

Gene[1]		Primer sequence (5'→3')	Accesion ID
CYP2E1	F	CGTGGAAATGGAGAAGGAAA	NM_000773.4
	R	GGTGATGAACCGCTGAATCT	
CAT	F	GAACTGTCCCTACCGTGCTCGA	NM_001752.4
	R	CCAGAATATTGGATGCTGTGCTCCAGG	
SOD	F	AATGGACCAGTGAAGGTGTGGGG	NM_000454.5
	R	CACATTGCCCAAGTCTCCAACATGC	
GPX	F	CGGCCCAGTCGGTGTATGC	NM_001329503.2
	R	CGTGGTGCCTCAGAGGGAC	
SREBP1c	F	CGGAGCCATGGATTGCACT	NM_001005291.3
	R	TAGGCCAGGGAAGTCACTG	
FAS	F	TCGTGGGCTACAGCATGGT	NM_004104.5
	R	GCCCTCTGAAGTCGAAGAAGAA	
PPARα	F	CAAACTTGGACCTGAACGAT	NM_001362873.2
	R	GAACGGTTTCCTTAGGCTTT	
CPT1	F	TTTCCTTGCTGAGGTGCTCT	NM_004377.4
	R	TCTCGCCTGCAATCATGTAG	
ACO	F	TCTGTTGACCTTGTTCGAGCAA	NM_001185039.2
	R	CAAGCACAGAGCCAAGTGTCAC	
GAPDH	F	ACCCACTCCTCCACCTTTG	NM_001357943.2
	R	CTCTTGTGCTCTTGCTGGG	

[1] *CYP2E1*, Cytochrome P450 2E1; *CAT*, catalase; *SOD*, superoxide dismutase; *GPX*, glutathione peroxidase; *SREBP1*, sterol regulatory element-binding protein 1; *FAS*, fatty acid synthase; *PPARA*, peroxisome proliferator-activated receptor alpha; *CPT1*, carnitine palmitoyltransferase 1; *ACO*, acyl-CoA 1; *GAPDH*, glyceraldehyde 3-phosphate dehydrogenase.

Table S2. The minimum inhibitory concentrations (MIC) cut-off value from EFSA.

**Antimicrobials**	Microbiological cut-off values (μg/mL)		
	L. brevis	*L. fermentum*	*L. reuteri*
Ampicillin	2	2	2
Gentamicin	16	16	8
Kanamycin	64	64	64
Streptomycin	64	64	64
Tetracycline	8	8	32
Chloramphenicol	4	4	4
Erythromycin	1	1	1
Vancomycin	n.r[1]	n.r.	n.r.
Clindamycin	4	4	4

[1] n.r., not required.

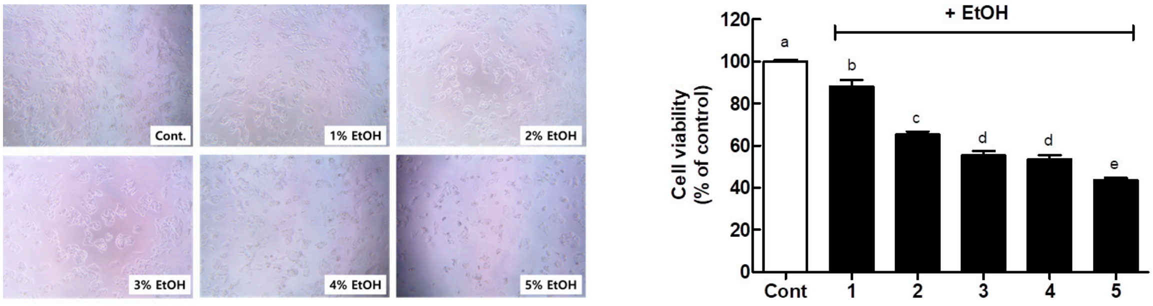

(a) **(b)**

Figure S1. The viability of ethanol (EtOH) on HepG2 cells. The cells were treated with various concentrations of EtOH l (1 - 5%) for 24 h. The data was expressed as the mean ± SD (n = 3). Different letters on the column meant significant at $p < 0.05$ based on Duncan's test.

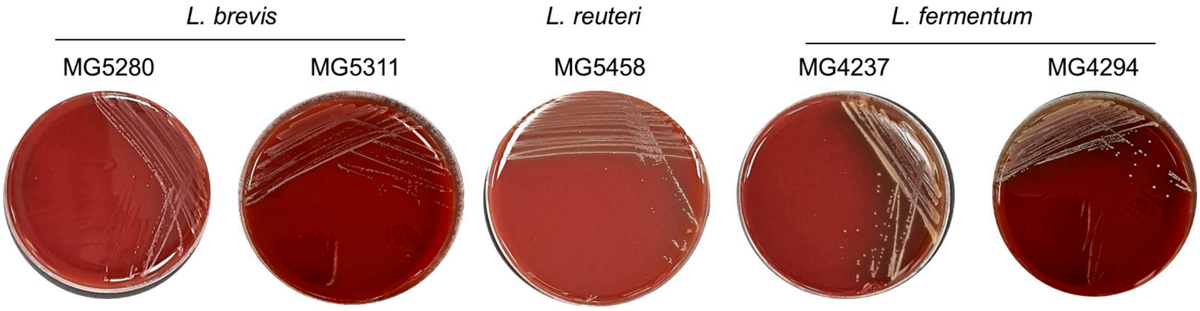

Figure S2. The hemolytic activity of LAB strains.
